# Using Voice Activity Detection and Deep Neural Networks with Hybrid Speech Feature Extraction for Deceptive Speech Detection †

**DOI:** 10.3390/s22031228

**Published:** 2022-02-06

**Authors:** Serban Mihalache, Dragos Burileanu

**Affiliations:** Speech and Dialogue Research Laboratory, University “Politehnica” of Bucharest, 060042 Bucharest, Romania; dragos.burileanu@upb.ro

**Keywords:** deceptive speech detection, deep neural networks, RODeCAR, voice activity detection

## Abstract

In this work, we first propose a deep neural network (DNN) system for the automatic detection of speech in audio signals, otherwise known as voice activity detection (VAD). Several DNN types were investigated, including multilayer perceptrons (MLPs), recurrent neural networks (RNNs), and convolutional neural networks (CNNs), with the best performance being obtained for the latter. Additional postprocessing techniques, i.e., hysteretic thresholding, minimum duration filtering, and bilateral extension, were employed in order to boost performance. The systems were trained and tested using several data subsets of the CENSREC-1-C database, with different simulated ambient noise conditions, and additional testing was performed on a different CENSREC-1-C data subset containing actual ambient noise, as well as on a subset of the TIMIT database. An accuracy of up to 99.13% was obtained for the CENSREC-1-C datasets, and 97.60% for the TIMIT dataset. We proceed to show how the final VAD system can be adapted and employed within an utterance-level deceptive speech detection (DSD) processing pipeline. The best DSD performance is achieved by a novel hybrid CNN-MLP network leveraging a fusion of algorithmically and automatically extracted speech features, and reaches an unweighted accuracy (UA) of 63.7% on the RLDD database, and 62.4% on the RODeCAR database.

## 1. Introduction

In recent years, the usage of spoken language to control or interact with devices and systems has become more and more prevalent. Ranging from virtual assistants and smart home devices to real-time speech translation, biometric identification, or forensic applications, such systems have been developed employing several techniques, with some of the best-performing ones using artificial intelligence (AI) models [1].

However, recorded audio signals include a variety of other types of content apart from speech: ambient noise, silence intervals, and other non-vocal content. It is often essential that, within the longer speech processing chain, speech features are only extracted from intervals containing actual speech data, so that the AI model is trained and tested only on relevant information. Thus, one of the first tasks that must be addressed within a speech processing pipeline is voice activity detection (VAD), i.e., the precise labeling of utterance time intervals within audio recordings.

In this work, we define *utterances* as the content of intervals of an audio signal in which speech is present, separated by pauses of at least 200 ms from other such intervals.

Successfully employed techniques use algorithmically extracted speech features such as energy, the zero-crossing rate (ZCR), the harmonic distribution and the harmonic-to-noise ratio, and perceptual linear prediction (PLP) coefficients or the mel-frequency cepstral coefficients (MFCCs), as well as various spectral features and their time derivatives, together with machine learning models including hidden Markov models (HMMs), Gaussian mixture models (GMMs), or hybrid GMM-HMM models [2,3,4,5], Kalman filters [6], or, more recently, neural networks: multilayer perceptrons (MLPs) [7,8] and recurrent neural networks (RNNs), particularly using long short-term memory (LSTM) cells in order to avoid the vanishing gradient problem [9,10]. More recently, deep learning models, especially convolutional neural networks (CNNs), have been employed together with automatic feature extraction, either directly from the time-domain samples, or using a frequency-domain representation of the signal, such as the discrete Fourier transform (DFT) or spectrograms [11]. Finally, various hybrid models have also led to promising results, e.g., jointly trained CNN-MLP or CNN-RNN models [12,13], but these come with a significant computational cost, especially if the VAD model is only one of several blocks within a larger speech processing system. Specific results previously reported in literature for the databases used in this work just for the VAD task, the Corpus and Environment for Noisy Speech Recognition (CENSREC-1-C) [14] and the TIMIT Acoustic-Phonetic Continuous Speech Corpus (TIMIT) [15], are provided in Section 3.3.

Additionally, certain prosodic features (e.g., speech onset latency, utterance duration, verbal interaction time, or pauses between utterances pertaining to the same speaker turn) are very important in paralinguistic applications [16]. Another particularly important case is deceptive speech detection (DSD), i.e., the ability to discern the truthfulness of a subject’s speech content, the potential deception involved being used for concealing incriminating information or misleading law enforcement agents during interviews.

While a system for lie detection from speech would not provide legal support (the well-known polygraph test is not admissible as scientific evidence in a court of law either), the results may prove to be of higher accuracy, since some speech characteristics are generally more difficult to alter voluntarily than conventionally tracked parameters (respiration rate, heart rate, blood pressure, etc.) [17], which can be manipulated into yielding false results [18]. Even though a multimodal approach would lead to greater performance, a lie detector based solely on audio data can be used inconspicuously in relevant scenarios (police interviews, checkpoint monitoring, etc.) so as to reduce the subject’s awareness, leading to a lower chance for the subject to manipulate the results.

Previous research on the topic of DSD often tackles the problem in the context of corpora that use actors or pretrained participants, such as the Columbia/SRI/Colorado corpus (CSC) [19] or the Columbia X-Cultural Deception corpus (CXD) [20], i.e., simulated behavior, in a relaxed, low-stakes environment, with the data having been annotated subjectively, constituting significant disadvantages, especially regarding their departure from the characteristics of such actual real-life interactions. Consequently, a better approach would involve using more realistic databases, in which participants have not received any information regarding their expected behavior, have full autonomy over the content and form of their answers, and are placed in (and aware of) high-stakes scenarios in which both engaging in deceptive behavior and admitting incriminating truths would lead to relatively severe consequences.

Two such publicly available corpora are the Real-Life Trial Data for Deception Detection (RLDD) database [21] and the Romanian Deva Criminal Investigation Audio Recordings (RODeCAR) database [22], the latter having been developed by the authors of this work. Apart from the previously mentioned characteristics, both databases involve data annotation being performed in a manner as objectively and conclusively as possible, by having additional information available after the data had been collected (judicial trial or criminal investigation outcomes) on which the annotation was based.

For the DSD task, previous research was carried out using traditional algorithmically extracted speech features or statistical descriptors derived from that, including the mean and standard deviation of the pitch [23], the MFCCs and their delta and double-delta coefficients [24,25], jitter, the harmonic-to-noise ratio (HNR) [26], or other acoustic and prosodic features [25,27] based on the ComParE feature set [28]. As for the machine learning models used, these include support vector machines (SVMs) [16,23,25,26], random forests (RFs) [20,23,25,27,29,30], MLPs [23,25,27,31], logistic regression [25,31], or ensemble methods with multiple classifiers and average/majority voting [27]. Specific results previously reported in literature for the databases used in this work for the DSD task, RLDD and RODeCAR, are provided in Section 3.5.

This article builds upon our previous work and is an improved and substantially extended version of our paper [32]. The main contributions of our work are:

1. Proposing, implementing, and validating a VAD system based on CNNs, using several postprocessing techniques, obtaining improved performance on the widely used benchmark database CENSREC-1-C, as well as on the TIMIT database;
2. Proposing, implementing, and validating a modified version of the VAD system, based on a hybrid CNN-MLP network, to be used for utterance-level DSD on the RLDD and RODeCAR databases;
3. Proposing, implementing, and validating a novel DSD system based on a hybrid CNN-MLP network and using a fusion of automatically and algorithmically extracted speech features, selected based on Kolmogorov–Smirnov feature ranking;
4. Obtaining results for the DSD task approached in a “*local lie*” manner, i.e., determining which particular utterances are *truthful* vs. *deceptive*, more relevant for forensic and law enforcement applications than other approaches (e.g., determining the overall *truthful* or *deceptive* attitude of a speaker).

The rest of the article is organized as follows. In Section 2, we discuss the proposed VAD system architecture and the proposed DSD system that incorporates it. In Section 3, we further describe the datasets used in this work, present the experimental setup, as well as the obtained results, and show how the proposed VAD system outperforms the previous state of the art and how it can be applied to the task of DSD. Finally, we draw conclusions and consider our intended future work in Section 4.

## 2. System Architecture

### 2.1. The Voice Activity Detection (VAD) System

In the first part of this work, we investigate several DNN types, i.e., MLPs, LSTM-based RNNs, and CNNs, together with three optimized postprocessing techniques: hysteretic thresholding, minimum duration filtering, and bilateral extension.

**Figure 1:**
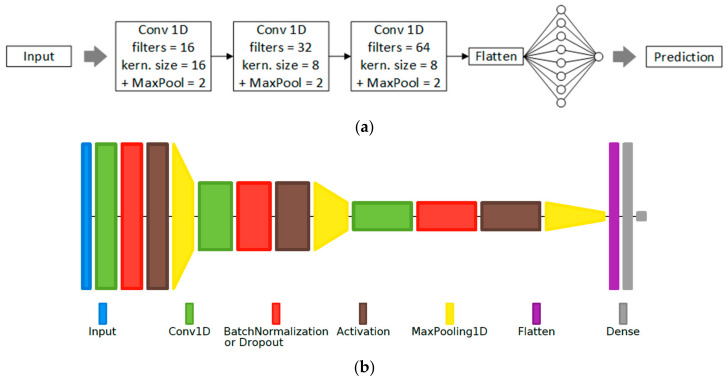
CNN-based VAD system. In this configuration example, the three convolutional (Conv) layers use 16, 32, and 64 filters, respectively, with corresponding kernel sizes of 16, 8, and 8. The size of the max-pooling layers used after the Conv layers is 2. The third Conv layer’s output is flattened into a one-dimensional vector and fed through a fully connected hidden layer with 8 nodes, used together with the output neuron with the “sigmoid” activation function for final frame-level classification. (**a**) Block diagram representation; (**b**) Layer diagram representation using Net2Vis [33].

The proposed MLP-based VAD systems use two hidden layers and are coupled with traditional algorithmically extracted features (the energy, the ZCR, the harmonic-to-noise ratio (HNR), the normalized autocorrelation coefficient, and the first 13 MFCCs) which are grouped into a number of feature sets. The RNN-based systems use an LSTM layer, followed by fully connected layers for the actual classification, and are coupled with the same feature sets mentioned previously. The CNN-based systems use 3 pairs of convolutional and max-pooling layers, followed by fully connected layers for classification; the raw time-domain samples or the frequency-domain representation of the signal, given by the 127-point Discrete Fourier Transform (DFT) (i.e., bins 1–127 of the 256-point DFT), are provided at the CNN input. An example of a proposed CNN structure is illustrated as a block diagram in Figure 1a, with the corresponding layer diagram shown in Figure 1b.

The input audio is first preprocessed (resampled, initially, at 8 kHz, and normalized in amplitude) and then split into overlapping frames, using Hamming windowing (the frame length and stride being among the hyperparameters tuned during validation).

The algorithmic features for the first two types of DNNs, as well as the extracted DFT for the latter type, are computed at the frame level and normalized by the z-score transform. These features (or the raw frame) are then used as the input to the DNN. The model’s output will represent the probability that the frames include speech content.

A sliding window encompassing 3 consecutive frames is used to average these probabilities and the resulting value is compared to a threshold. If the value is above the threshold, the window will be considered *positive* (containing speech). Consecutive *positive* windows determine the utterance start and stop times.

**Figure 2:**
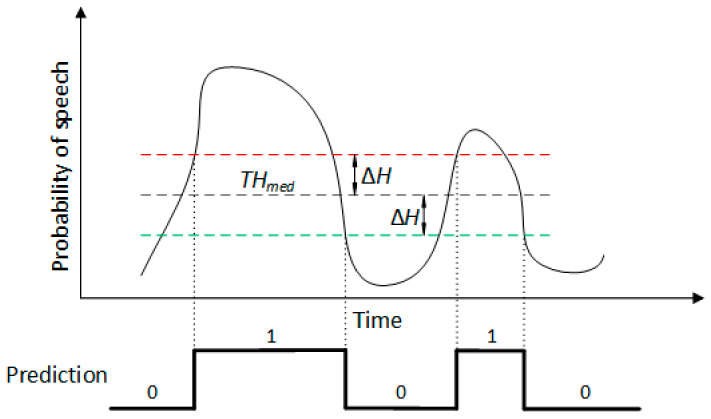
Hysteretic thresholding. For a window to be considered *positive* after the previous ones had been *negative*, the probability must be greater than *TH_med_* + ∆*H*. Similarly, for the opposite case, the threshold is *TH_med_* − ∆*H*.

**Figure 3:**
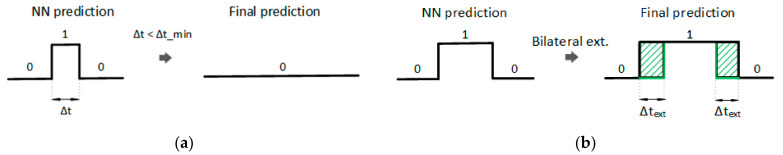
(**a**) Minimum duration filtering; if a resulting utterance has a shorter duration than a reference value, ∆*t_min_*, it is discarded; (**b**) Bilateral extension; the utterance start time is lowered by a value ∆*t_ext_*, while its stop time is increased by the same value.

In order to boost performance, we do not employ a single threshold, but use hysteresis to obtain two separate ones, the higher one being used when switching from *negative* to *positive* predictions, and the lower one being used in the opposite case. This principle is illustrated in Figure 2, where *TH_med_* is the median threshold and ∆*H* is the hysteresis size. Additionally, if a resulting utterance has a shorter duration than a reference value, ∆*t_min_*, it is discarded, as shown in Figure 3a. For the remaining predicted utterances, we also use a bilateral extension of their durations to compensate for the system’s tendency to underestimate the utterance length, i.e., the utterance start time is lowered by a value ∆*t_ext_*, while its stop time is increased by the same value, as shown in Figure 3b. These four hyperparameters (*TH_med_*, ∆*H*, ∆*t_min_*, and ∆*t_ext_*) are optimized during validation.

**Figure 4:**
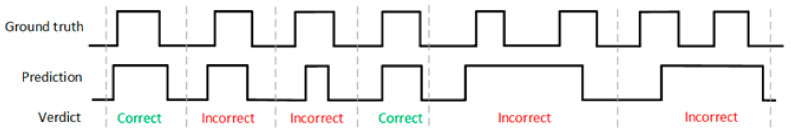
Layer diagram representation of the final hybrid CNN-MLP-based VAD system, using the same configuration example as in Figure 1.

Utterance-level predictions are considered correct if there’s a perfect match to the ground truth time stamps or if they are slightly extended on either side of the true utterance, without including a subsequent utterance, even partially, as can be seen in Figure 4.

**Figure 5:**
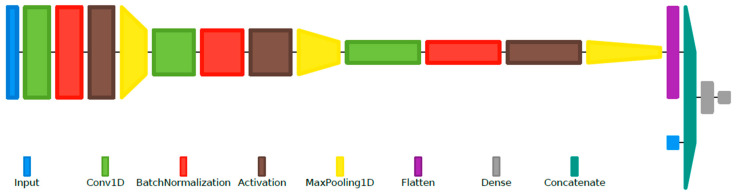
Layer diagram representation of the final hybrid CNN-MLP-based VAD system, using the same configuration example as in Figure 1.

To adapt the VAD system developed using the CENSREC-1-C and the TIMIT corpora for the DSD databases (RLDD and RODeCAR), a number of adjustments were made. First, the system, with the architecture presented before, was retrained on a small part of the new datasets to account for the differences in ambient noise type and level and recording quality. Further, taking advantage of the higher sampling rate available for the DSD databases, 16 kHz, the frequency-domain input was taken as a 255-point DFT, corresponding to the same frequency resolution as the initial case. Finally, a modified hybrid CNN-MLP network was implemented and tested, using as additional input the first 13 MFCCs and their delta and double-delta coefficients (39 features), extracted for each frame with the same frame length and stride. These additional algorithmically extracted features are concatenated with the ones automatically extracted by the convolutional layers, after the flattening layer. This final, modified VAD system architecture is presented in Figure 5.

### 2.2. The Deceptive Speech Detection (DSD) System

**Figure 6:**
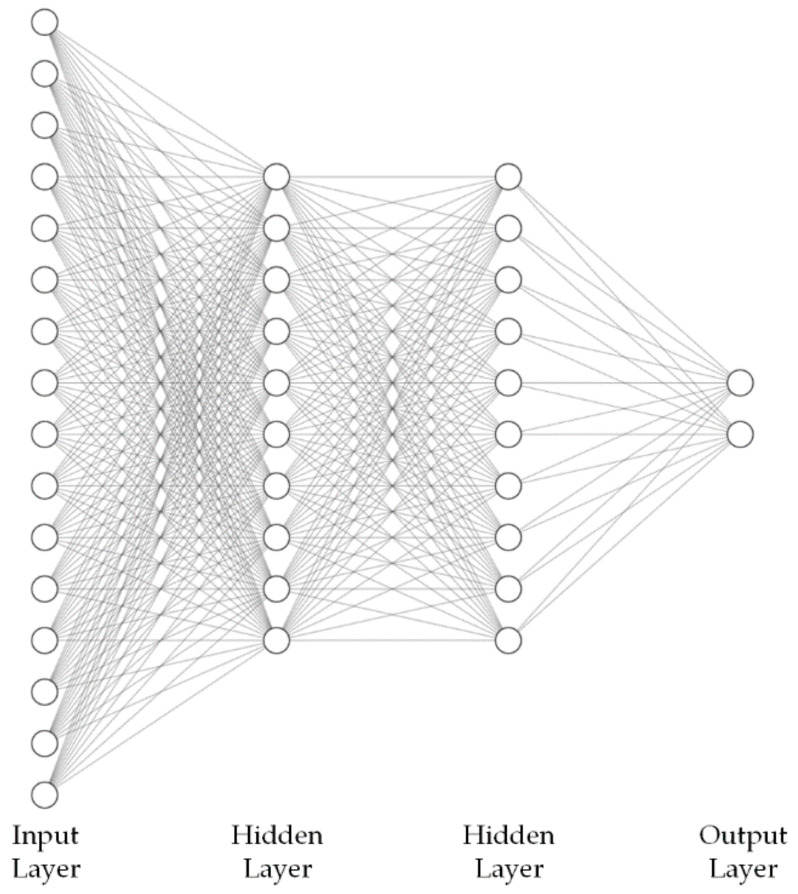
Layer diagram representation of the basic DSD MLP-based system, using between 2 and 3 hidden layers with 64 or 128 nodes per layer, and with an output layer of size 2, the number of classes taken into account (*truthful* and *deceptive*), and applying the “softmax” activation function.

The basic proposed deep learning system, illustrated in Figure 6, consists of a deep neural network (DNN) taking as input an extensive set of descriptors obtained by applying high level statistical functions on algorithmically extracted acoustic, prosodic, spectral, and cepstral features. The DNN classifier is a multilayer perceptron (MLP) model, using between 2 and 3 hidden layers with 64 or 128 nodes per layer, but with an output layer whose size is equal to the number of classes taken into account, i.e., 2 (*truthful* and *deceptive*), and uses the “softmax” activation function, instead of a single neuron applying the “sigmoid” activation function. This configuration for the output layer was chosen empirically after observing that having a separate output probability of the sample belonging to each class allowed the optimization algorithm to better adjust the DNN weights than having a single output probability of the sample belonging to the *deceptive* class.

**Figure 7:**
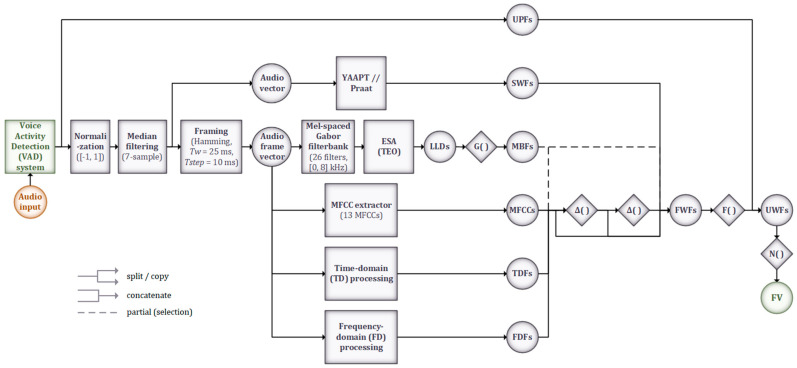
Detailed block diagram representation of the preprocessing and feature extraction stages of the DSD system. The VAD system is used to detect and split the input audio into utterances from each of which the set of 2260 features is extracted.

The preprocessing and feature extraction stages are detailed in Figure 7. The former consists of applying amplitude normalization and 7-sample median filtering to each utterance detected by the VAD system, as well as framing the signal using Hamming windows of 25 ms duration with a 15 ms overlap. The feature set used is an extension of the ComParE feature set [28], and also includes the modulation-based features (MBFs) proposed in [34] and utilized successfully in our previous work on speech emotion recognition [35], as well as two utterance-wise prosodic features (UPFs): utterance duration and leading pause duration, i.e., the time interval between the end of the previous utterance and the start of the current one, both shown as relevant for the DSD task [19,20].

The Python implementations of the Yet Another Algorithm for Pitch Tracking (YAAPT) algorithm [36] and Praat [37] are used to extract segment-wise features (SWFs), with the same stride employed for framing the input audio (10 ms): pitch, harmonic-to-noise ratio (HNR), local jitter and local shimmer. The other features are extracted frame-wise and consist of: (i) the MBFs, obtained through applying a 26-filter Mel-spaced Gabor filterbank and the Teager Energy Separation Algorithm (ESA-TEO) [34], followed by the G(·) set of functionals (mean, standard deviation, weighted mean, weighted squared bandwidth) on the resulting low-level descriptors (LLDs); (ii) the first 13 MFCCs; (iii) time-domain features (TDFs); and (iv) frequency-domain features (FDFs). The TDFs are RMS energy and zero-crossing rate (ZCR). The FDFs are low-frequency energy (250–650 Hz), high-frequency energy (1–4 kHz), spectral flux, spectral centroids, spectral spread, spectral skewness, spectral kurtosis, spectral entropy, spectral slope, spectral roll-off points (25%, 50%, 75%, 90%), and the log-filterbank energies. Delta and double-delta coefficients are computed for the MFCCs, TDFs, and FDFs, as well as for some of the MBFs. Together with the SWFs, these represent the frame-wise features (FWFs), on which the F(·) set of functionals (mean and standard deviation) is applied. These are then combined with the UPFs noted previously, resulting in the utterance-wise features (UWFs).

The N(·) function, z-score normalization, is applied per speaker. The obtained normalized feature vector (FV) has a total size of 2260 and is subsequently fed to the DNN classifiers in its entirety or using various subsets, as detailed in Section 3.4.

**Figure 8:**
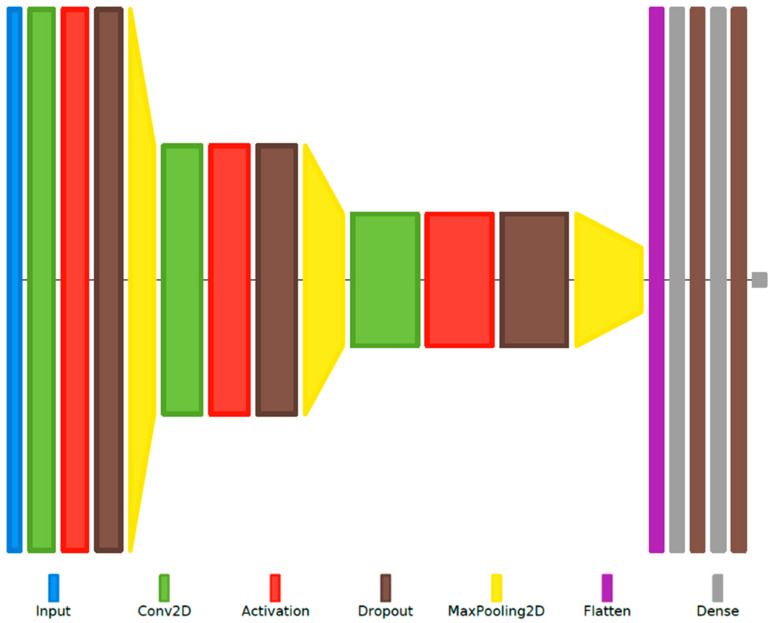
Layer diagram representation of the CNN-based DSD system. In this configuration example, the three convolutional (Conv) layers use 16, 32, and 64 filters, respectively, with kernel sizes of 3 × 3. The size of the max-pooling layers used after the Conv layers is 2 × 2. The third Conv layer’s output is flattened into a one-dimensional vector and fed through two fully connected hidden layers with 32 nodes each, used together with the output layer with the “softmax” activation function for utterance-level classification.

A second DSD system is proposed, leveraging the nature of automatic feature extraction offered by CNNs. Illustrated in Figure 8, the model takes as input the magnitude spectrogram of each preprocessed utterance, extracted using Hamming windows of 25 ms duration with a 15 ms overlap, scaled linearly into 257 frequency bins (corresponding to half the sampling frequency), and zero-padded to the duration (in number of frames) of the longest utterance in the dataset. Subsequently, three stages of 2D convolutional layers are applied with a small and constant receptive field, with max-pooling applied after each one to reduce the dimensionality of the data. The output of the final pooling layer represents the set of automatically extracted feature maps that are then flattened into a one-dimensional vector and passed through a sequence of fully connected hidden layers. Together with the output layer, these form the actual classifier stages, similar to the basic DNN system discussed at the beginning of this section, and adopting a configuration (number of layers, number of nodes per layer) according to the best structure determined previously.

Finally, in order to boost performance further, a final hybrid CNN-MLP network is proposed for this task as well, combining the automatically extracted features provided by the convolutional stages with the best subset of algorithmically extracted features determined using the basic DNN-based DSD system. These features, extracted at the utterance level as described beforehand, are provided as an additional input, and are concatenated with the output of the flattening layer before being fed to the classifier (MLP) stage of the hybrid network.

## 3. Experimental Setup and Results

For the VAD task, training and testing were conducted using several data subsets of the Corpus and Environment for Noisy Speech Recognition (CENSREC-1-C) [14], most often used as a benchmark for such applications, and a subset of the TIMIT Acoustic-Phonetic Continuous Speech Corpus (TIMIT) [15].

We also used the Real-Life Trial Data for Deception Detection (RLDD) database [21] and the Romanian Deva Criminal Investigation Audio Recordings (RODeCAR) database [22]: in their original structure (as entire audio recordings) for the VAD task, and, for the DSD task, as datasets aggregating all valid speech utterances available in the respective recordings (i.e., not belonging to interviewers—prosecutors, lawyers, etc.)

All proposed systems were developed in Keras, a neural networks framework for Python, running over the TensorFlow backend. All experiments were performed on a Linux machine running Ubuntu 18.04, with a 16 core Intel Xeon E5 2600 CPU at 3.20 GHz, 192 GB of RAM at 2133 MHz, and an 8 GB Nvidia Quadro M4000 GPU.

### 3.1. Databases and Subsets

#### 3.1.1. The CENSREC-1-C Database

The CENSREC-1-C database consists of recordings of eleven Japanese digits, uttered by native speakers for different simulated and actual noise environments. There are two sets of simulated noise environments, A and B, each consisting of four types of additive ambient noise (A = subway, babble, car, and exhibition; B = restaurant, street, airport, and station), added at different signal-to-noise ratios (SNRs): clean (>30 dB), 20 dB, 15 dB, 10 dB, 5 dB, 0 dB, and −5 dB. For each noise type, the number of speakers is 104 (52 female, 52 male). The database also includes files recorded in two actual noise environments (in a restaurant and near a highway), each under two SNR levels (high and low) using 10 speakers (5 female, 5 male). All files use an 8 kHz sample rate and the 16-bit PCM format.

In our experiments, we use set A as is, but split set B into B1 and B2 (B1 = airport and station; B2 = restaurant and street) and label the files recorded in actual noise conditions as set R; total audio content: A = 22 h, B1 = 8 h, B2 = 11 h, R = 80 min.

#### 3.1.2. The TIMIT Database

The TIMIT database contains 6300 utterances, made up of 10 sentences spoken by 630 speakers (192 female, 438 male) using different English dialects encountered throughout the United States. All original files were recorded without ambient noise (SNR > 30 dB) and stored at a 16 kHz sample rate. The total size of the corpus is 5 h. We used a subset of 433 recordings, with a total duration of 24 min, for additional testing.

#### 3.1.3. The RLDD Database

The RLDD database comprises 121 audiovisual recordings in English of defendants and witnesses, obtained from famous trials conducted in the United States. The guilty or non-guilty verdicts and, in some cases, the exoneration of previously incarcerated individuals, allowed for the objective annotation of the data into 61 recordings labeled as *deceptive* and 60 recordings labeled as *truthful*. The content duration totals 56 min, with an average recording length of 28 s. Excluding prosecutors, lawyers, and other interviewers, the total number of speakers is 56 (22 female, 34 male).

The audio tracks were extracted using the FFmpeg framework and saved in 16-bit PCM format, at 16 kHz sampling rate. In order to be able to retrain the VAD system on this database as mentioned at the end of Section 2.1, we manually annotated the start and stop times of each utterance within each audio recording.

#### 3.1.4. The RODeCAR Database

The RODeCAR database was constructed using media files acquired in 9 older criminal cases, investigating murder, sexual assault, and fraud charges. After an initial filtering of the content, all 20 involved speakers (4 female, 16 male) and the prosecutors were manually identified (ID) and associated with an ID number, with special values reserved for the prosecutors (to facilitate the exclusion of their associated content from the database). The audio tracks were extracted using the FFmpeg framework and saved in 16-bit PCM format, at 16 kHz sampling rate.

For each of the 26 resulting audio recordings, we employed semi-automatic annotation into utterances defined as portions of speech pertaining to a single speaker, either (i) separated by a pause of at least 200 ms from other portions of speech from the same speaker, or (ii) separated from other portions of speech from a different speaker, regardless of the delay duration. For ambiguous cases (e.g., the prosecutor and the participant cutting each other off), the stop time of the interviewer’s utterance is chosen at the end of the last phone in their speech, coinciding with the start time of the participant’s utterance, i.e., considering a null speech onset latency.

In order to determine the truthfulness of the participants’ statements as clearly and accurately as possible, a meticulous manual review of the recordings and associated case notes was conducted together with the prosecutor who originally conducted the investigations. The binary annotation (*truthful*/*deceptive*) is made per utterance, but in a global sense, e.g., a short utterance, containing factually accurate information, found between longer utterances in which the behavior is deceptive, will also be labeled as *deceptive*. This is further supported by the psychological argument that the state of mind in which the participants find themselves when lying will be sustained by the long-term goal of deceiving the prosecutor, the deception cues still being present in the participants’ speech.

The database consists of 7 h 32 min of total content, of which 4 h 46 min represent actual speech content. Out of this, 3 h 27 min represent the participants’ utterances (excluding those pertaining to the prosecutors); 2 h 6 min represent the *truthful* speech content, while 1 h 21 min represent the *deceptive* speech content. The *truthful* content comprises 60.5% of the total speech content (excluding the prosecutors’ speech), with the other 39.5% being labeled as *deceptive*.

### 3.2. Setup and Details–VAD Task

The first proposed system (MLP-based) uses two hidden layers (with 8 and 4 nodes, respectively) with “ReLU” activation and a single output node with “sigmoid” activation. Dropout between 0.5 and 0.8 was also used after each hidden layer. The second system (RNN-based) uses a single LSTM layer (with an output feature vector size of 32) and a dropout of 0.5. After preliminary testing using different numbers of convolutional layers and their parameters, the third system (CNN-based) was configured as per Figure 1, either employing dropout between 0.5 and 0.7 or a batch normalization layer [38], used before each pooling layer for better regularization (both cannot be used simultaneously due to the internal covariance shift [39]).

Three schemes were used for framing the audio recordings: (i) 25 ms length, 15 ms overlap; (ii) 50 ms length, 10 ms overlap; (iii) 30 ms length, 5 ms overlap. For the algorithmically extracted features, after preliminary testing with different feature groupings, we determined that the best results were obtained when using only the MFCCs for the RNN model and the MFCCs and the energy for the MLP model, these being the features used in subsequent experiments for those models. For the postprocessing hyperparameters, the median threshold was taken between 0.3 and 0.9, ∆*H* between 0.0 and 0.2, ∆*t_min_* between 25 ms and 125 ms, and ∆*t_ext_* between 125 ms and 750 ms.

For the preliminary tests described, as well as for investigating the frame-level performance of the models, we used the B1 subset (training on 70% and validating on 30%). For the postprocessing hyperparameter optimization and final results (utterance-level performance), we used the B2 subset with the same training–validation split. Then, we tested the performance on the A and R subsets, as well as on the TIMIT subset.

After benchmarking the best performing VAD system developed on the CENSREC-1-C and the TIMIT corpora against the DSD databases (RLDD and RODeCAR), we adapted it through retraining, without changing its configuration, on a small part of the new datasets. The corresponding audio recordings were preprocessed applying the same framing scheme, resulting in 135,523 audio frames for the RLDD dataset (105,076 containing speech and thus being labeled *positive*; and 30,447 being labeled *negative*) and 1,129,450 audio frames for the RODeCAR dataset (691,365 *positive*; and 438,085 *negative*). The retraining was performed using 10-fold cross-validation, with 10% of the data reserved for training and 90% for validation, an acceptable approach as it would considerably reduce the time required to adapt the system to a new corpus by manually annotating it in its entirety.

The same experiments were repeated after modifying the frequency-domain input as a 255-point DFT, taking advantage of the 16 kHz sampling rate available for the DSD corpora, and corresponding to the same frequency resolution as the 127-point DFT at 8 kHz.

Finally, the hybrid CNN-MLP network was implemented and tested using the same methodology, adding as a secondary input the algorithmically extracted features discussed in Section 2.1, obtained frame-wise with the same frame length and overlap.

The metrics used are the accuracy, precision, false acceptance rate (FAR), i.e., the rate of false *positives*, and false rejection rate (FRR), i.e., the rate of false *negatives*. These metrics are defined in Equations (1)–(4), where TP, TN, FP, and FN represent the number of true *positives*, true *negatives*, false *positives*, and false *negatives*, respectively.

(1) $$\mathrm{Accuracy}=\frac{\mathrm{TP}+\mathrm{TN}}{\mathrm{TP}+\mathrm{FN}+\mathrm{TN}+\mathrm{FP}}$$

(2) $$\mathrm{Precision}=\frac{\mathrm{TP}}{\mathrm{TP}+\mathrm{FP}}$$

(3) $$\mathrm{FAR}=\frac{\mathrm{FP}}{\mathrm{TN}+\mathrm{FP}}$$

(4) $$\mathrm{FRR}=\frac{\mathrm{FN}}{\mathrm{TP}+\mathrm{FN}}$$

### 3.3. Results and Discussions–VAD Task

Unless otherwise specified, the results will be given for framing scheme 3 (30 ms length, 5 ms overlap) and for the frequency-domain (DFT) input in the case of CNN models (using time-domain input did not achieve relevant performance). The frame-level cross-validation results (before the postprocessing stage) for the three DNN model types are presented in Table 1. A threshold value of 0.5 was used. As can be observed, the best results are achieved with the CNN model. Additionally, while the FRR does appear to have a prohibitively large value, this is only due to the fact that the CENSREC-1-C files contain long continuous time intervals labeled as *positive*, even despite obvious pauses in speech ranging up to a few hundreds of milliseconds in duration.

Next, we investigated how the CNN results are influenced by the median threshold, *TH_med_*, but found that no performance gain could be achieved. Instead, we employed the hysteretic thresholding technique and obtained the best results using ∆*H* = 0.1.

**Figure 9:**
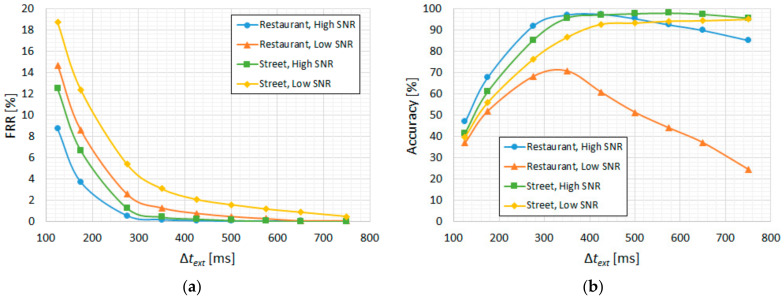
CENSREC-1-C subset B2; CNN-based VAD. “High SNR” groups the 10–20 dB levels and “Low SNR” groups the −5–5 dB levels: (**a**) FRR vs. ∆*t_ext_*; (**b**) accuracy vs. ∆*t_ext_*.

**Figure 10:**
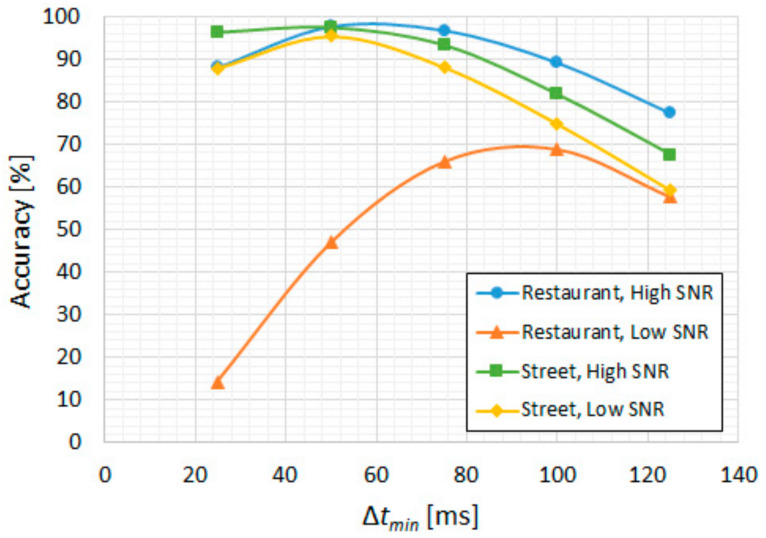
CENSREC-1-C subset B2; CNN-based VAD. “High SNR” groups the 10–20 dB levels and “Low SNR” groups the −5–5 dB levels: accuracy vs. ∆*t_min_*.

For the bilateral extension (∆*t_ext_*), we illustrate the results in Figure 9a in terms of FRR and in Figure 9b in terms of accuracy. We observe a considerable reduction in the utterance-level FRR (miss rate) up to around 350 ms, which also coincides with the best accuracy for these ambient noise types, thus giving an optimal ∆*t_ext_* = 350 ms. Similarly, for minimum duration filtering, since the other three ambient noise types are more relevant than low-SNR “restaurant”, the resulting optimal ∆*t_min_* is 50 ms, as can be seen in Figure 10.

With the postprocessing hyperparameters selected as described, additional testing was conducted on subset A (Japanese) and on the TIMIT subset (English), in order to ensure better generalization. The results for subset A are presented in Table 2. The best results are obtained, naturally, for the clean recordings, reaching an accuracy of 98.2%. Additionally, we can observe a dramatic decrease in performance for low SNR levels (below 10 dB). For the TIMIT subset, a similar accuracy of 97.6% was achieved.

Final testing at this stage was conducted on CENSREC-1-C subset R (real ambient noise), with detailed results being shown in Table 3, alongside those reported in other works in literature. The number at the end of the proposed CNN-based systems (1, 2, 3) refers to the framing scheme used during audio input preprocessing. The highlighted system (CNNs with DFT input and 30 ms frames with 5 ms overlap) offers the best results for all conditions, with the sole exception of the highway ambient noise type in [6], because the latter is better suited for noises with a large frequency spread.

Selecting the best-performing VAD system (CNN-based, framing scheme 3), the frame-level cross-validation results obtained on the RLDD and RODeCAR databases are presented in Table 4. The benchmark refers to its direct evaluation, without retraining, and with the configuration described previously; this configuration led to poor performance at the utterance level, after applying the postprocessing techniques, justifying the approaches considered further. Consistent improvements can be seen for each version, i.e., retraining the system without changing its configuration, retraining it with the frequency-domain input expanded to the 255-point DFT (16 kHz sampling rate), and retraining it with the modified hybrid CNN-MLP network architecture using the additional 39 cepstral features described in Section 2.1.

**Figure 11:**
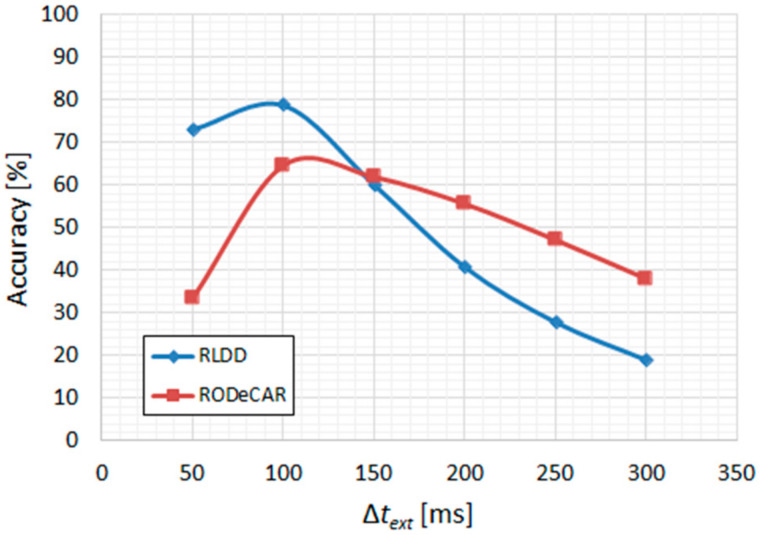
RLDD and RODeCAR; final CNN-MLP-based VAD, with expanded frequency-domain input (255-pt. DFT at 16 kHz sampling rate), retrained on 10% of the data: accuracy vs. ∆*t_ext_*.

With the latter configuration chosen as the final VAD system, the postprocessing hyperparameters were optimized similarly as before, keeping the median threshold, *TH_med_*, equal to 0.5, but reducing the hysteresis size to ∆*H* = 0.05. The minimum duration, ∆*t_min_*, was also kept equal to 50 ms. The bilateral extension parameter, ∆*t_ext_*, had to be considerably reduced due to the much shorter pauses between utterances specific to the two new datasets. As can be observed in Figure 11, the optimal value in both cases was 100 ms.

The utterance-level performance, after choosing the optimal hyperparameters, is given in Table 5. The relatively low accuracy, similar to the corresponding results obtained in Table 3, is due to the nature of the audio content having been recorded in real-life environments, a large number of them having low SNR (especially in the case of the RODeCAR database) and, in many cases, containing ambient noise similar to the “restaurant” and “babble” types encountered in the CENSREC-1-C database. Further possible improvements, e.g., including a speech enhancement stage after the preprocessing block, are reserved for future work, and are discussed in Section 4.

### 3.4. Setup and Details–DSD Task

Other research previously published concerning the RLDD database for DSD uses a speaker-level [23] or recording-level approach [24,26,27]; the former involves determining the overall *truthful* or *deceptive* attitude of each speaker (56 instances), while the latter attempts to classify each audio recording (121 total instances) in its entirety as *truthful* or *deceptive*. For the first case, the approach used in [23] reaches an accuracy of 61.0% using an MLP-based system and 52 audio features, and 71.2% using an RF of 100 trees with only the standard deviation of the pitch as input. For the second case, described in [24], the reported accuracy is 81.5% for the entire set of 121 files; in [26], only 100 of the 121 instances are used, and the performance using only audio features reaches a low accuracy of 34.2%; and, applying ensemble classification with majority voting, the authors of [27] report an accuracy of 70.0%.

However, neither the speaker-level nor the recording-level approach is best suited for forensic and law enforcement applications, as targeted by this work, since the purpose is instead to determine when (and regarding what) a subject is likely lying throughout an interaction of arbitrary duration. Thus, we adopt a different and more challenging “*local lie*” (utterance-level) approach, determining which particular utterances in each recording are *truthful* vs. *deceptive*; this difference invalidates a comparison to the other approaches. To this end, for the DSD task, all utterances were separately extracted from each audio recording available in the RLDD and RODeCAR corpora, totaling 931 (467 *truthful* and 464 *deceptive*) and 5859 (3136 *truthful* and 2723 *deceptive*), respectively.

Unless otherwise specified, for all DSD experiments, 10-fold cross-validation with speaker separation was employed as the testing methodology, with an 80%/20% training-validation split, ensuring the same ratio of *truthful* and *deceptive* samples are available in each subset, as well as having the same ratio of male and female speakers.

The basic MLP-based models were evaluated for a depth (number of hidden layers) between 2 and 3, with 64 or 128 nodes per hidden layer. Other hyperparameters chosen include: the “ReLU” activation function for the hidden layers; “Adam” [40] as the optimization algorithm; L1-norm regularization with the regularization parameter equal to 10^−4^. The batch size was set to 32. The experiments were run for up to 100 epochs, with early stopping and learning rate decay (between 10^−3^ and 10^−6^, with a decay factor of 0.1). Since the RODeCAR database is slightly imbalanced in terms of class distribution (53.5% of the utterances are *truthful*), class weighting was employed, boosting the contribution of the *deceptive* samples in computing the loss function by their representation ratio (i.e., 1.152).

The basic systems were tested for the total feature set of size 2260 described in Section 2.2, as well as for several feature subsets. First, 4 homogenous groups are obtained, applying the F(·) set of functionals and normalizing using the N(·) function, for the following features: (i) SWFs + TDFs, with the group subsequently labeled as acoustic-prosodic features (APF); (ii) FDFs, labeled as FDF; (iii) MFCCs, labeled as MFCC; (iv) MBFs, labeled as MBF. A fifth group consists of just the normalized UPFs and is labeled as UPF. Then, every possible combination of 2, 3, and 4 homogenous groups (10, 10, and 5 combinations, respectively) is tested. Finally, using a proposed feature selection algorithm based on the two-sample Kolmogorov–Smirnov test (KS) [41], an additional 5 subsets were obtained by selecting the most relevant 10, 20, 50, 100, and 200 features, in terms of the KS statistic, resulting in a total number of 36 feature subsets.

The proposed feature selection algorithm involves the following steps:

1. For each feature in the full set of size 2260, establish the null hypothesis that its distribution for the *truthful* samples is the same as that for the *deceptive* samples (i.e., the feature would not be relevant for the DSD task);
2. Compute the empirical cumulative distribution functions (ECDFs) of each feature, according to Equation (5), where: *F_k,Nk_*(*x*) are the ECDFs across all values for the *truthful* samples (*k* = 0) and for the *deceptive* samples (*k* = 1), respectively, with *N_k_* representing the number of samples in the class; 1_[–∞,*x*]_(*X_i_*) is the indicator function, equal to 1 if *X_i_* ≤ *x* or 0 otherwise; and *X_i_* is the current sample;
3. Compute the KS statistic, D, using the two ECDFs and Equation (6), where sup is the supremum function;
4. Eliminate the features for which the null hypothesis cannot be rejected with *p* = 0.01, i.e., when Equation (7), derived from the KS critical value tables [42], does not hold;
5. Rank the remaining (relevant) features in reverse order according to the value of D;
6. Select the top features according to the desired number.

(5) $$F_{{k,N}_{k}}\left(x\right)=\frac{1}{N_{k}}\overset{N_{k}}{\underset{i=1}{\sum }}1_{\left[–\infty ,x\right]}\left(X_{i}\right),\;k\;\in \;\left\{0,\;1\right\}$$

(6) $$D=\underset{x}{\sup}\left|F_{{0,N}_{0}}\left(x\right){\;-\;F}_{{1,N}_{1}}\left(x\right)\right|$$

(7) $$D\;>\;1.628\cdot \sqrt{\frac{N_{0}{+N}_{1}}{N_{0}\cdot N_{1}}}$$

The top 10 features extracted using this algorithm, together with their D values, are listed for the two databases in Table 6 and Table 7. For reference, the corresponding 26-filter Mel-spaced Gabor filterbank central frequencies and bandwidths are given in Table 8.

For the other two proposed DSD systems, based on a CNN model or using a hybrid CNN-MLP network, the input spectrograms must be of the same size, thus requiring zero-padding to the duration (in number of frames) of the longest utterance in each database. For the framing scheme described in Section 2.2 (25 ms duration with 15 ms overlap), this results in 1183 frames (spectrogram width) for RLDD, and 2392 for RODeCAR. Additionally, a dropout of 0.4 was used before each pooling layer, and L2-norm regularization was employed instead. The MLP stage consists of two fully connected layers, with 32 nodes per layer. All other applicable hyperparameters follow the same configuration as for the basic DSD system described previously. Due to the large number of model parameters required by these systems (over 20 million), 3-fold cross-validation was employed instead to reduce the training and validation time.

In order to account for the imbalance in the class distribution present in the RODeCAR database, both the unweighted accuracy (UA) and the weighted accuracy (WA) are used as performance metrics, with the UA being more robust since it represents the average of the accuracies of the predictions made for each class (or, equivalently, the average recall rate). The WA is the same as the accuracy used as a metric in the VAD experiments and defined in Equation (1), but it is rewritten according to Equation (8), where *K* = 2 is the number of classes, *H_k_* is the number of correct predictions made for class *k*, and *N* = *N*_0_ + *N*_1_ is the total number of samples. It can easily be seen that TP and TN from Equation (1) represent the same as *H*_0_ and *H*_1_, and that *N* = TP + TN + FP + FN. The UA is defined in Equation (9).

(8) $$\mathrm{WA}=\frac{1}{N}\overset{1}{\underset{k=0}{\sum }}H_{k}=\frac{1}{K}\overset{1}{\underset{k=0}{\sum }}\frac{K\cdot N_{k}}{N}\cdot \frac{H_{k}}{N_{k}}$$

(9) $$\mathrm{UA}=\frac{1}{K}\overset{1}{\underset{k=0}{\sum }}\frac{H_{k}}{N_{k}}$$

### 3.5. Results and Discussions–DSD Task

For the basic MLP-based DSD systems, the best-performing configuration comprised 2 hidden layers with 64 nodes per layer. For the RLDD dataset, the best-feature subset consisted of the top 10 features selected by the proposed KS-based algorithm, yielding a UA of 59.3%; all features were part of the FDF group. For the RODeCAR dataset, the combined APF + MBF + UPF subset led to the highest UA, 54.9%. Further investigation is required to explain why none of the top selected features using the KS-based algorithm determined better performance. All test cases are reported in Table 9.

The results for the CNN-based DSD system are given in Table 10. As can be observed, the automatically extracted feature maps provided by the convolutional layers allow for better separation in their distribution between the two classes, leading to a relative performance increase over the basic systems of 5.56% for the RLDD dataset and 11.84% for the RODeCAR dataset in terms of UA.

For the final hybrid CNN-MLP-based DSD system, the results are shown in Table 11. The combined feature maps provided by the convolutional layers and the best-performing feature subset determined in the previous experiments lead to a further increase in relative performance of 1.76% and 1.63% for the RLDD and RODeCAR datasets, respectively, demonstrating the effectiveness of the approach of using a fusion of automatically and algorithmically extracted features as inputs to a deep learning model for the DSD task.

As a final note regarding model complexity, in terms of size, the MLP models (which employs algorithmically extracted features) reach up to 323,000 parameters (150,000 for the best performing configuration of 2 hidden layers, 64 nodes per layer, and the set of all features), whereas the CNN and CNN-MLP models (which include automatic feature extraction) reach up to 20,000,000 parameters. In terms of the input complexity, there are up to 2260 algorithmically extracted features (for the MLP models) vs. 64 automatically extracted feature maps of size up to 299 × 33 (depending on the maximum utterance length within the database), i.e., up to 630,000 descriptors (for the CNN model), vs. the combination of the feature maps and the highest-rated feature subsets. However, despite the much higher complexity of the CNN and CNN-MLP models, in the MLP-based DSD experiments, the best results are obtained using only subsets of the 2260 features (of size 10 or 340), as was shown in Table 9, suggesting that a larger set of algorithmically extracted features would not serve to reach better performance. Rather, the automatically extracted feature maps better capture the relevant deception-related information encoded in the frequency-domain representations of the audio signals, with the best performance being obtained with the hybrid CNN-MLP model, as was shown in Table 10 and Table 11.

## 4. Conclusions

We have proposed, implemented, and validated several voice activity detection (VAD) systems based on DNNs and using three postprocessing techniques: hysteretic thresholding, minimum duration filtering, and bilateral extension. With the CNN-based system, we have obtained improved performance on the widely used benchmark database CENSREC-1-C (up to 99.13% accuracy), as well as on the TIMIT database (up to 97.60% accuracy). With the final hybrid CNN-MLP-based network, after adapting it for use with the RLDD and RODeCAR datasets, we have shown how the VAD system can be incorporated within the speech processing pipeline for deceptive speech detection (DSD) by being used to split each input audio recording into utterances, allowing for a new, different, and more difficult utterance-level approach to the DSD task. Additionally, the VAD system is also employed for the extraction of utterance-wise prosodic features such as the utterance duration and the leading pause duration.

The utterance-level (“*local lie*”) approach, i.e., determining which particular utterances are *truthful* vs. *deceptive*, is better suited than other speaker-level approaches (i.e., determining the overall *truthful* or *deceptive* attitude of a speaker) or recording-level approaches (i.e., determining the overall *truthful* or *deceptive* character of an entire speaker interaction of arbitrary duration) for forensic and law enforcement applications (e.g., police interviews, checkpoint monitoring, etc.), especially if the subject’s awareness is reduced, leading to a lower likelihood for them to manipulate the results.

For this approach, we have proposed, implemented, and validated several DSD systems. The highest-performing architecture was a novel hybrid CNN-MLP-based network using a fusion of automatically extracted feature maps from the linear magnitude spectrogram as the frequency-domain input, and subsets of algorithmically extracted features, selected based on a proposed feature selection algorithm employing two-sample Kolmogorov–Smirnov feature ranking. The system reaches an accuracy of 63.7% on the RLDD database, and 62.4% on the RODeCAR database. Due to the different nature and scale of our approach, a comparison to the results presented in Section 3.4, obtained by other works on the RLDD corpus, cannot be made. At the same time, this work represents the first published results for the RODeCAR database.

For future work, we intend to investigate other deep learning models for the VAD task, as well as including a speech enhancement stage in the processing pipeline in order to obtain a higher SNR and reduce the impact of the ambient noise. For the DSD task, we intend to investigate additional prosodic features (e.g., speech rate, onset latency, etc.) and alternative feature selection algorithms, as well as to include an attention mechanism for the convolutional layers in order to allow for a larger receptive field by increasing the kernel size without increasing the computational cost.

## Figures and Tables

**Table 1:** VAD frame-level cross-validation performance vs. model type–CENSREC-1-C subset B1.

Metric	Model		
	MLP	RNN	CNN
Accuracy [%]	77.07	79.87	**80.25**
Precision [%]	68.59	76.65	**85.50**
FAR [%]	11.65	7.67	**5.60**
FRR [%]	46.75	46.39	49.10

**Table 2:** CNN-based VAD utterance-level test accuracy [%] vs. SNR–CENSREC-1-C subset A.

SNR	Ambient Noise Type			
	Subway	Babble	Car	Exhibition
>30 dB	**98.20**			
20 dB	94.80	96.10	96.28	96.60
15 dB	93.41	91.60	92.28	92.40
10 dB	92.40	90.90	90.17	91.70
5 dB	87.61	86.51	87.47	88.71
0 dB	78.40	56.74	82.16	74.02
−5 dB	60.50	22.37	67.33	68.13

**Table 3:** VAD utterance-level top test accuracy [%] vs. model type–CENSREC-1-C subset R.

System	Ambient Noise Type					Model	Descriptors
	Restaurant		Highway		Average		
	High SNR	Low SNR	High SNR	Low SNR			
CENSREC-1-C baseline [14]	74.20	56.50	39.40	41.40	52.88	HMM	MFCC, Energy
[5]	76.75	63.02	92.44	79.64	77.96	GMM-HMM	Energy, Pitch, DFT
**[6]**	**92.75**	**65.51**	**100.00**	**100.00**	**89.57**	**Kalman**	**MFCC**
[8]	75.65	21.45	95.94	49.86	60.73	MLP	MFCC
MLP	88.11	57.46	56.65	54.34	64.14	MLP	MFCC, Energy
RNN	74.25	39.10	65.80	51.50	57.66	RNN (LSTM)	MFCC
CNN-DFT1	85.21	64.92	82.60	75.65	77.10	CNN	DFT
CNN-DFT2	97.10	59.13	95.36	89.56	85.29	CNN	DFT
CNN-DFT3	**99.13**	**68.69**	**97.97**	**90.72**	**89.13**	**CNN**	**DFT**

**Table 4:** VAD frame-level cross-validation performance vs. model type–RLDD and RODeCAR.

Metric	Model							
	Original CNN 127-pt. DFT, 8 kHz s.r. No Retraining (Baseline)		Original CNN 127-pt. DFT, 8 kHz s.r. Retrained (10%)		Original CNN 255-pt. DFT, 16 kHz s.r. Retrained (10%)		CNN-MLP 255-pt. DFT, 16 kHz s.r. Retrained (10%)	
	RLDD	RODeCAR	RLDD	RODeCAR	RLDD	RODeCAR	RLDD	RODeCAR
Accuracy [%]	83.22	80.55	86.18	85.92	91.53	86.37	**94.92**	**89.92**
Precision [%]	89.55	87.34	–	–	–	–	**96.79**	**90.89**
FAR [%]	35.73	18.25	–	–	–	–	**11.05**	**14.69**
FRR [%]	11.31	20.21	–	–	–	–	**3.35**	**7.15**

**Table 5:** Final CNN-MLP-based VAD utterance-level test performance–RLDD and RODeCAR.

Model	Accuracy [%]	
	RLDD	RODeCAR
CNN-MLP; input: 255-pt. DFT + 39; 16 kHz s.r.	**78.71**	**64.45**

**Table 6:** Top 10 selected features using the proposed KS-based algorithm–RLDD.

Feature	Description	In Group	D
m_d_Sxx_EgyH	Mean of delta of high frequency energy (1–4 kHz)	FDF	0.300252
m_d_Sxx_Enpy_sbb_14	Mean of delta of spectral entropy in subband 14	FDF	0.269789
m_d_Sxx_Enpy_sbb_6	Mean of delta of spectral entropy in subband 6	FDF	0.261999
m_d_Sxx_Slop_sbb_3	Mean of delta of spectral slope in subband 3	FDF	0.252644
m_d_Sxx_Slop_sbb_15	Mean of delta of spectral slope in subband 15	FDF	0.250614
m_d_Sxx_Slop_sbb_0	Mean of delta of spectral slope in the full band (0–8 kHz)	FDF	0.250545
m_d_Sxx_Slop_sbb_2	Mean of delta of spectral slope in subband 2	FDF	0.250102
m_Sxx_Enpy_sbb_2	Mean of spectral entropy in subband 2	FDF	0.248943
m_d_Sxx_Enpy_sbb_13	Mean of delta of spectral entropy in subband 13	FDF	0.248888
m_d_Sxx_EgyL	Mean of delta of low frequency energy (250–650 Hz)	FDF	0.248805

**Table 7:** Top 10 selected features using the proposed KS-based algorithm–RODeCAR.

Feature	Description	In Group	D
m_dd_Sxx_Roll75_sbb_1	Mean of double delta of 75% spectral roll-off in subband 1	FDF	0.231245
m_d_Sxx_Roll90_sbb_1	Mean of delta of 90% spectral roll-off in subband 1	FDF	0.230591
s_dd_Sxx_Skew_sbb_10	Std. dev. of double delta of spectral skewness in subband 10	FDF	0.228881
s_dd_Sxx_Skew_sbb_11	Std. dev. of double delta of spectral skewness in subband 11	FDF	0.221929
s_d_Sxx_Skew_sbb_10	Std. dev. of delta of spectral skewness in subband 10	FDF	0.220162
s_d_Sxx_Skew_sbb_11	Std. dev. of delta of spectral skewness in subband 11	FDF	0.217571
m_dd_Sxx_Enpy_sbb_23	Mean of double delta of spectral entropy in subband 23	FDF	0.213800
m_d_Sxx_Slop_sbb_1	Mean of delta of spectral slope in subband 1	FDF	0.211873
m_dd_Sxx_Enpy_sbb_22	Mean of double delta of spectral entropy in subband 22	FDF	0.205927
m_d_Sxx_Kurt_sbb_3	Mean of delta of spectral kurtosis in subband 3	FDF	0.196904

**Table 8:** Central frequencies and bandwidths for the 26-filter Mel-spaced Gabor filterbank.

Subband	Central Freq. [Hz]	Bandwidth [Hz]	Subband	Central Freq. [Hz]	Bandwidth [Hz]
1	62.5	125	14	1890.5	469
2	140	156	15	2140.5	531
3	218.5	187	16	2421.5	593
4	312	188	17	2718.5	625
5	406	188	18	3062	688
6	515.5	219	19	3421.5	781
7	625	250	20	3828	844
8	765.5	281	21	4281	938
9	906	312	22	4765.5	1031
10	1078	344	23	5312.5	1125
11	1249.5	375	24	5906	1250
12	1453	406	25	6546.5	1343
13	1656	438	26	7265.5	1469

**Table 9:** MLP-based DSD cross-validation performance vs. feature subsets–RLDD and RODeCAR.

Feature Subset	Subset Size	RLDD		RODeCAR	
		UA [%]	WA [%]	UA [%]	WA [%]
all	2260	54.0	54.0	51.0	53.4
APF	26	53.0	53.0	52.4	53.4
FDF	1842	55.3	55.3	52.4	52.9
MFCC	78	52.0	52.0	52.5	52.9
MBF	312	58.6	58.6	53.8	54.8
UPF	2	49.8	49.7	51.4	**59.6**
APF + FDF	1868	55.5	55.5	52.7	52.8
APF + MFCC	104	52.5	52.5	53.6	54.2
APF + MBF	338	57.5	57.5	53.9	54.2
APF + UPF	28	51.8	51.8	52.6	53.0
FDF + MFCC	1920	54.9	54.9	52.6	53.2
FDF + MBF	2154	56.5	56.5	52.8	52.1
FDF + UPF	1844	55.1	55.2	51.9	52.5
MFCC + MBF	390	57.9	57.9	52.7	55.0
MFCC + UPF	80	53.4	53.4	52.0	53.6
MBF + UPF	314	56.8	56.8	53.4	55.5
APF + FDF + MFCC	1946	55.7	55.7	53.1	54.7
APF + FDF + MBF	2180	56.0	56.0	51.6	51.3
APF + FDF + UPF	1870	57.4	57.5	52.4	53.5
APF + MFCC + MBF	416	57.4	57.5	53.8	54.6
APF + MFCC + UPF	106	54.8	54.8	52.4	53.4
**APF + MBF + UPF**	**340**	58.1	58.1	**54.9**	55.0
FDF + MFCC + MBF	2232	57.2	57.2	51.6	52.4
FDF + MFCC + UPF	1922	54.9	55.0	52.4	54.0
FDF + MBF + UPF	2156	55.5	55.5	52.7	53.8
MFCC + MBF + UPF	392	55.4	55.4	53.1	55.1
APF + FDF + MFCC + MBF	2258	56.8	56.8	52.1	54.7
APF + FDF + MFCC + UPF	1948	55.7	55.7	52.0	54.2
APF + FDF + MBF + UPF	2182	55.5	55.5	52.1	53.6
APF + MFCC + MBF + UPF	418	56.0	56.0	53.0	55.6
FDF + MFCC + MBF + UPF	2234	56.2	56.2	52.3	51.7
**top-10 selected by the KS-based algorithm**	**10**	**59.3**	**59.2**	51.6	53.6
top-20 selected by the KS-based algorithm	20	58.1	58.2	52.3	56.5
top-50 selected by the KS-based algorithm	50	58.3	58.3	52.5	53.3
top-100 selected by the KS-based algorithm	100	58.0	58.0	52.0	53.4
top-200 selected by the KS-based algorithm	200	55.0	55.1	52.3	51.5

**Table 10:** CNN-based DSD cross-validation performance–RLDD and RODeCAR.

Model	RLDD		RODeCAR	
	UA [%]	WA [%]	UA [%]	WA [%]
CNN; input: 1183 × 257/2392 × 257 linear magnitude spectrogram; 16 kHz s.r.	62.6	62.6	61.4	61.6

**Table 11:** Final CNN-MLP-based DSD cross-validation performance–RLDD and RODeCAR.

Model	RLDD		RODeCAR	
	UA [%]	WA [%]	UA [%]	WA [%]
CNN-MLP; input: 1183 × 257/2392 × 257 linear magnitude spectrogram + 10/340; 16 kHz s.r.	**63.7**	**63.6**	**62.4**	**62.5**

## Data Availability

The RODeCAR database is available on request from the corresponding author of this paper, and can be provided on demand using the instructions found here: https://speed.pub.ro/downloads/paralinguistic-datasets/.

## References

[1] Hinton G. (2012). Deep Neural Networks for Acoustic Modeling in Speech Recognition: The Shared Views of Four Research Groups. IEEE Signal Process. Mag..

[2] Shao Y., Lin Q. Use of Pitch Continuity for Robust Speech Activity Detection. Proceedings of the IEEE International Conference on Acoustics, Speech and Signal Processing (ICASSP).

[3] Wisdom S. Voice Activity Detection Using Subband Noncircularity. Proceedings of the IEEE International Conference on Acoustics, Speech and Signal Processing (ICASSP).

[4] Heese F., Niermann M., Vary P. Speech-codebook Based Soft Voice Activity Detection. Proceedings of the IEEE International Conference on Acoustics, Speech and Signal Processing (ICASSP).

[5] Espi M. Using Spectral Fluctuation of Speech in Multi-feature HMM-based Voice Activity Detection. Proceedings of the INTERSPEECH.

[6] Fujimoto M., Watanabe S., Nakatani T. Voice Activity Detection Using Frame-Wise Model Re-Estimation Method Based on Gaussian Pruning with Weight Normalization. Proceedings of the INTERSPEECH.

[7] Jassim W., Harte N. Voice Activity Detection Using Neurograms. Proceedings of the IEEE International Conference on Acoustics, Speech and Signal Processing (ICASSP).

[8] Fujimura H. Simultaneous Gender Classification and Voice Activity Detection Using Deep Neural Networks. Proceedings of the INTERSPEECH.

[9] Bai L., Zhang Z., Hu J. (2017). Voice Activity Detection Based on Deep Neural Networks and Viterbi. IOP Conf. Ser. Mater. Sci. Eng..

[10] Hughes T., Mierle K. Recurrent Neural Networks for Voice Activity Detection. Proceedings of the IEEE International Conference on Acoustics, Speech and Signal Processing (ICASSP).

[11] Ghaemmaghami H., Baker B., Vogt R., Sridharan S. Noise Robust Voice Activity Detection Using Features Extracted from the Time-domain Autocorrelation Function. Proceedings of the INTERSPEECH.

[12] Zazo Candil R., Sainath T.N., Simko G., Parada C. Feature Learning with Raw-Waveform CLDNNs for Voice Activity Detection. Proceedings of the INTERSPEECH.

[13] Thomas S., Saon G., Van Segbroeck M., Narayanan S.S. Improvements to the IBM Speech Activity Detection System for the DARPA RATS Program. Proceedings of the IEEE International Conference on Acoustics, Speech and Signal Processing (ICASSP).

[14] Kitaoka N., Yamamoto K., Kusamizu T. Development of VAD Evaluation Framework CENSREC-1-C and Investigation of Relationship between VAD and Speech Recognition Performance. Proceedings of the IEEE Workshop on Automatic Speech Recognition & Understanding (ASRU).

[15] Garofolo J.S. (1993). DARPA TIMIT Acoustic-Phonetic Continuous Speech Corpus.

[16] Montacie C., Caraty M.-J. Prosodic Cues and Answer Type Detection for the Deception Sub-Challenge. Proceedings of the INTERSPEECH.

[17] National Research Council (2003). The Polygraph and Lie Detection. Committee to Review the Scientific Evidence on the Polygraph. Division of Behavioral and Social Sciences and Education.

[18] Verschuere B., Prati V., De Houwer J. (2009). Cheating the Lie Detector. J. Psychol. Sci..

[19] Hirschberg J. Distinguishing Deceptive from Non-deceptive Speech. Proceedings of the INTERSPEECH.

[20] Levitan S.I. Cross-cultural Production and Detection of Deception from Speech. Proceedings of the ACM on Workshop on Multimodal Deception Detection.

[21] Perez-Rosas V., Abouelenien M., Mihalcea R., Burzo M. Deception Detection using Real-Life Trial Data. Proceedings of the ACM on International Conference on Multimodal Interaction.

[22] Mihalache S., Pop G., Burileanu D. Introducing the RODeCAR Database for Deceptive Speech Detection. Proceedings of the 10th International Conference on Speech Technology and Human-Computer Dialogue (SpeD).

[23] Sen U.M., Perez-Rosas V., Yanikoglu B., Abouelenien M., Burzo M., Mihalcea R. (2020). Multimodal Deception Detection using Real-Life Trial Data. IEEE Trans. Affect. Comput..

[24] Fathima Bareeda E.P., Shajee Mohan B.S., Ahammed Muneer K.V. (2021). Lie Detection using Speech Processing Techniques. J. Phys. Conf. Ser..

[25] Mendels G., Levitan S.I., Lee K.-Z., Hirschberg J. Hybrid Acoustic-Lexical Deep Learning Approach for Deception Detection. Proceedings of the INTERSPEECH.

[26] Jaiswal M., Tabibu S., Bajpai R. The Truth and Nothing but the Truth: Multimodal Analysis for Deception Detection. Proceedings of the IEEE 16th International Conference on Data Mining Workshops (ICDMW).

[27] Velichko A., Budkov V., Kagirov I., Karpov A., Kotenko I., Badica C., Desnitsky V., El Baz D., Ivanovic M. (2019). Applying Ensemble Learning Techniques and Neural Networks to Deceptive and Truthful Information Detection Task in the Flow of Speech. Intelligent Distributed Computing XIII. Studies in Computational Intelligence.

[28] Schuller B., Steidl S., Batliner A., Hirschberg J., Burgoon J.K., Baird A., Elkins A., Zhang Y., Coutinho E., Evanini K. The INTERSPEECH 2014 Computational Paralinguistics Challenge: Cognitive & Physical Load. Proceedings of the INTERSPEECH.

[29] Zhang J., Levitan S.I., Hirschberg J. Multimodal Deception Detection using Automatically Extracted Acoustic, Visual, and Lexical Features. Proceedings of the INTERSPEECH.

[30] Levitan S.I., Maredia A., Hirschberg J. Linguistic Cues to Deception and Perceived Deception in Interview Dialogues. Proceedings of the Conference of the North American Chapter of the Association for Computational Linguistics (NAACL).

[31] Kopev D., Ali A., Koychev I., Nakov P. Detecting Deception in Political Debates Using Acoustic and Textual Features. Proceedings of the IEEE Automatic Speech Recognition and Understanding Workshop (ASRU).

[32] Mihalache S., Ivanov I.-A., Burileanu D. Deep Neural Networks for Voice Activity Detection. Proceedings of the 44th International Conference on Telecommunications and Signal Processing (TSP).

[33] Bauerle A., Van Onzenoodt C., Ropinski T. (2021). Net2Vis—A Visual Grammar for Automatically Generating Publication-Tailored CNN Architecture Visualizations. IEEE Trans. Vis. Comput. Graph..

[34] Chaspari T., Dimitriadis D., Maragos P. Emotion Classification of Speech Using Modulation Features. Proceedings of the 22nd European Signal Proceedings Conference (EUSIPCO).

[35] Mihalache S., Burileanu D., Pop G., Burileanu C. Modulation-based Speech Emotion Recognition with Reconstruction Error Feature Expansion. Proceedings of the 10th International Conference on Speech Technology and Human-Computer Dialogue (SpeD).

[36] Kasi K., Zahorian S.A. Yet Another Algorithm for Pitch Tracking. Proceedings of the IEEE International Conference on Acoustics, Speech, and Signal Processing (ICASSP).

[37] Jadoul Y., Thompson B., De Boer B. (2018). Introducing Parselmouth: A Python Interface to Praat. J. Phon..

[38] Ioffe S., Szegedy C. Batch Normalization: Accelerating Deep Network Training by Reducing Internal Covariate Shift. Proceedings of the 32nd International Conference on Machine Learning.

[39] Li X., Chen S., Hu X., Yang J. Understanding the Disharmony between Dropout and Batch Normalization by Variance Shift. Proceedings of the IEEE/CVF Conference on Computer Vision and Pattern Recognition (CVPR).

[40] Kingma D.P., Ba J.L. Adam: A Method for Stochastic Optimization. Proceedings of the 3rd International Conference on Learning Representations (ICLR).

[41] Hodges J.L. (1958). The Significance Probability of the Smirnov Two-sample Test. Ark. Mat..

[42] Smirnov N. (1948). Table for Estimating the Goodness of Fit for Empirical Distributions. Ann. Math. Stat..

